# Alternatively spliced *MEFV* transcript lacking exon 2 and its protein isoform pyrin-2d implies an epigenetic regulation of the gene in inflammatory cell culture models

**DOI:** 10.1590/1678-4685-GMB-2016-0234

**Published:** 2017-08-31

**Authors:** Gokce Celikyapi Erdem, Sule Erdemir, Irem Abaci, Asli K. Kirectepe Aydin, Elif Everest, Eda Tahir Turanli

**Affiliations:** 1 Department of Molecular Biology Genetics and Biotechnology, Dr. Orhan Ocalgiray Molecular Biology and Genetics Research Centre, Graduate School of Science, Engineering and Technology, Istanbul Technical University, Istanbul, Turkey.; 2 Molecular Biology and Genetics Department, Faculty of Science and Letters, Istanbul Technical University, Istanbul, Turkey.

**Keywords:** Exon methylation, MEFV, pyrin, alternative splicing, inflammation

## Abstract

The function of gene body DNA methylation in alternative splicing, and its relation to disease pathogenesis is not fully elucidated. The gene for familial Mediterranean fever (*MEFV*) encodes the pyrin protein and contains a 998 bp CpG island, covering the second exon, which is differentially methylated in FMF patients compared to healthy controls. Our further observation of increased exon 2-spliced *MEFV* transcript in leukocytes of FMF patients provoked us to test the role of exon methylation in alternative splicing using inflammatory cell culture models. First, *in vitro* exon methylation triggered an increased level of exon 2 exclusion using a splicing cassette in a promyelocytic leukemia cell line (HL-60). HL-60 cells subjected to methylating and demethylating agents, as well as cells differentiated to neutrophil-like cells, exhibited different levels of spliced/unspliced transcripts. We observed increased levels of spliced transcripts in neutrophil-like (p = 0.0005), activated (p = 0.0034) and methylated cells (p < 0.0001), whereas decreased levels in demethylated cells (p = 0.0126) compared to control untreated HL-60 cells. We also showed that the protein isoform of pyrin lacking the exon 2 has an adverse subcellular localization in neutrophil-like cells. Therefore, it remains in the cytoplasm rather than the nucleus. This may point to an epigenetic involvement in an important inflammatory gene.

## Introduction

DNA methylation reduces gene expression by either blocking transcription start sites or interacting with nucleosomes that leads to heterochromatinization. Studies also show that promoter silencing precedes DNA methylation, which acts as a “lock” rather than a “silencer” (Lock *et al.*, 1987; Ohm *et al.*, 2007; Schlesinger *et al.*, 2007; Widschwendter *et al.*, 2007; Gal-Yam *et al.*, 2008). DNA methylation can also enhance gene expression through alternative promoters or by blocking insulators (reviewed in Jones, 2012). More recently, multiple sources of evidence positively correlate exon methylation with active gene transcription, which is also conserved across species (Jones, 1999; Nguyen *et al.*, 2001; Hellman and Chess, 2007; Cokus *et al.*, 2008; Hodges *et al.*, 2009; Feng *et al.*, 2010; Gonzalez *et al.*, 2016).

Furthermore, the link between exonic DNA methylation and alternative mRNA splicing through chromatin regulation (reviewed in Lev Maor *et al.*, 2015) has become better understood. Various studies performed with diverse cells and/or tissues from diverse sources show that alternate exons exhibit differential methylation patterns, being either abundantly or weakly methylated (Gelfman *et al.*, 2013; Wan *et al.*, 2013; Gutierrez-Arcelus *et al.*, 2015; Vujic *et al.*, 2015). Moreover, studies have demonstrated that the interruption of DNA methylation like DNA methyltransferase 3 (DNMT3) blockage/deletion (Li-Byarlay *et al.*, 2013; Vujic *et al.*, 2015) or 5-aza-2'-deoxycytidine treatment (Yang *et al.*, 2014) altered the splicing pattern.

Two possible mechanisms were proposed for the explanation of the effect of exonic DNA methylation on alternative splicing: a) methylation interferes with RNA Pol II elongation (Shukla *et al.*, 2011; Maunakea *et al.*, 2013), and b) recruitment of splicing factors to methylated sites (Saint-André *et al.*, 2011; Yearim *et al.*, 2015). Argonaute proteins (1 and 2), generally known for their role in transcriptional silencing, combine these two mechanisms by recruiting splice factors as well as slowing down the RNA Pol II elongation rate in a study which used CD44 gene as a model (Ameyar-Zazoua *et al.*, 2012).

However, a more specific indication of methylation in alternative splicing and chromatin dynamics, and its relation to disease pathogenesis is limited. MEFV contains a 998 bp CpG island (NC_000016.10 from 3254057 to 3255054) encompassing its whole exon 2. We have previously observed a negative correlation between methylation and expression levels of MEVF gene transcripts in familial Mediterranean fever (FMF) patients and control groups, in which patients were showing slightly higher exon 2 methylation levels (p = 0.049) (Kirectepe *et al.*, 2011a). Interestingly, exon 2 spliced transcript (*MEFV*-d2) levels were significantly higher in leukocytes of FMF patients compared to healthy controls (p = 0.026) (Kirectepe *et al.*, 2011b). This finding is compatible with the general notion that gene body methylation is positively correlated with expression (Yang *et al.*, 2014).

*MEFV* (MEditerranean FeVer) is the gene responsible for FMF, which is an autoinflammatory disease characterized by acute episodes of inflammation, with a high incidence in Mediterranean populations. It is suggested that pathogenic variants on *MEFV* gene result in defective pyrin production, which in turn affects FMF pathology (The International FMF Consortium, 1997). However, there are certain percentages of FMF patients (5–15%), depending on the ethnic background, who do not carry *MEFV* pathogenic variants but still present a full FMF phenotype (Lidar and Livneh, 2007). *MEFV* protein product pyrin is known to have a regulatory role in inflammation as part of the inflammasome complex. *MEFV* is mainly expressed in neutrophils, eosinophils, monocytes, dendritic cells and synovial fibroblasts (Centola *et al.*, 2000) and its expression is increased by proinflammatory agents such as interferon γ (IFN-γ), tumor necrosis factor α (TNF-α), lipopolysaccharide (LPS) and interleukin 1β (IL-1β) (Matzner *et al.*, 2000).

Although *MEFV* is generally transcribed into a major full-length transcript, 14 alternatively spliced transcripts are known, and among those only six get translated into protein isoforms; d2, d2/8ext, d2/9ext, 8ext, 2a, 2a/4a (Grandemange *et al.*, 2009, Medlej-Hashim *et al.*, 2010). Pyrin-d2, which is the first described pyrin isoform, is generated by in-frame alternative splicing of exon 2 and expressed in peripheral blood leukocytes (PBLs) (Papin *et al.*, 2000). The subcellular localization of full-length pyrin and its d2 isoform were investigated in several cell lines. Full-length pyrin (pyrin-fl) is cytoplasmic and d2 isoform is mainly nuclear (Papin *et al.*, 2000; Tidow *et al*.; 2000, Cazeneuve *et al.*, 2003). As an exception, one study indicated that myc-tagged d2 was not exclusively nuclear and was regularly cytoplasmic in synovial fibroblasts, suggesting that it may shuttle between cytoplasm and nucleus (Diaz *et al.*, 2004). Nonetheless, native pyrin, which consists predominantly of pyrin-fl, was nuclear in synovial fibroblasts, neutrophils, and dendritic cells, but was cytoplasmic in monocytes. Moreover, the localization of pyrin-fl and pyrin-d2 was not affected by the most frequent *MEFV* pathogenic variants (Cazeneuve *et al.*, 2003).

Here, we aimed to analyze the possible relationship between splicing of *MEFV* exon 2 and its methylation using *in vitro* cell culture model systems to further investigate our *in vivo* results from FMF patients (Kirectepe *et al.*, 2011a,b). HL-60 promyelotic cells were first transfected with methylated and non-methylated splicing constructs using a splicing cassette, as a preliminary *in vitro* study to assess the possible role of methylation on the alternative splicing of *MEFV* second exon. Later, expression levels of the exon 2 lacking transcripts were analyzed in cell culture models, using methanol as methylating and 5-aza-2'deoxycytidine as demethylating agents, DMSO for differentiation to neutrophil-like cells, and LPS as an activating agent. Methylation status analysis of cell culture systems was also performed using real-time quantitative PCR analysis, which allowed us to explore the methylation level of *MEFV* CpG island. We have shown that *in vitro* methylation of the splicing cassette containing the second exon of *MEFV* leads to its splicing. We also observed that *MEFV*-d2 transcript levels were increased when cells were subjected to methylation, differentiated to neutrophil-like cells or activated, and decreased when the cells were demethylated.

Because abnormal localization of proteins participates in the pathogenesis of many human diseases (Hung and Link, 2011; Agostinho *et al.*, 2015; Liu and Hu, 2016), we also studied localization differences of pyrin-fl and pyrin-d2 exploring the localization of recombinant constructs via confocal microscopy. Our results also confirmed that pyrin full-length form was localized in cytoplasm and exon 2 spliced form in nucleus of HL-60 cell-line. On the other hand, unlike previous findings, both forms were found to be localized in the cytoplasm of neutrophil-like cells. Our results showed for the first time that methylation causes splicing of the second exon, which leads to the inability of pyrin-d2 form to localize into nucleus in neutrophil-like cells. These findings strengthen our hypothesis of *MEFV-*d2 transcripts having a role in inflammatory conditions through epigenetic modifications.

## Material and Methods

### Splicing reporter assay using pSpliceExpress cassette

The insert sequence comprised in the CpG island (800 bp), starting from NC_000016.10 3254236 until 3255036, which covers the whole of exon 2 and 85 nucleotides from intron 1 and 83 nucleotides from intron 2 was amplified from peripheral blood genomic DNAs of healthy control samples without any pathogenic or benign *MEFV* variations via PCR, using the primers given in Supplementary Table S1.

A second PCR reaction was performed to add the appropriate recombination sites (attb 1 and 2) with primers given in supplementary Table S2. Amplicons were cloned to pSpliceExpress (Kishore *et al.*, 2008) vector using amplicon Gateway® BP Clonase® II Enzyme (Invitrogen, Waltham, Massachusetts, USA), and the recombination product was transformed to *E. coli* Top 10 cells via heat-shock. After overnight incubation, plasmid isolation was performed from colonies using High Pure Plasmid Isolation Kit (Roche Diagnostics, Mannheim, Germany), followed by measurement of the plasmid concentrations using a Nanodrop (Thermo Fisher Scientific Inc., Waltham, MA USA) spectrophotometer. Later, half of the amount was methylated with CpG Methylase (M. SssI) (Zymo Research, Irvine, CA, USA) overnight at 30 °C, and the other half was left unmethylated. The methylation of the insert was confirmed with digestion using SmaI enzyme, which cuts at non-methylated CCC/GGG sites.

HL-60 cells were cultured in RPMI 1640 medium containing 10% FBS and 300 μL penicillin/streptomycin. Then the cells (2 x 10^6^) were transfected with methylated and unmethylated pSpliceExpress cassettes containing the CpG island DNA element (2 μg), together with the empty pSpliceExpress vector as a negative control, by nucleofection using Amaxa® Cell Line Nucleofector® Kit V (Amaxa, Cologne, Germany). Transfected cells were incubated for 24 h at 37 °C in a humidified atmosphere containing 5% CO_2_, and RNA isolation was performed using High Pure RNA Isolation Kit (Roche Diagnostics, Mannheim, Germany). cDNA synthesis was done using High Capacity cDNA Reverse Transcription Kit (Applied Biosystems, Inc. Foster City, California). PCR reaction was setup with rat insulin primers (given in supplementary Table S3), which are specific to the rat insulin exons present within the pSpliceExpress vector, known to be concurrently spliced.

### Cell culture models

HL-60 promyelotic cells were cultured in liquid suspension in RPMI 1640 medium supplemented with 10% fetal bovine serum, 2 mM L-glutamine, 100 U/mL penicillin and 100 pg/mL streptomycin (each from Lonza, Amaxa, Cologne, Germany). The cells were cultured at 37 °C in a humidified atmosphere containing 5% CO_2_. Different cell culture models were generated to mimic different *in vivo* conditions as explained below.

#### a) Neutrophil-like cells

Cells were induced to differentiate into neutrophils with 1.75% (vol/vol) DMSO for 6 days. 2 x 10^5^ cells/mL, 1.5 x 10^5^ cells/mL, 1 x 10^5^ cells/mL and 0.5 x 10^5^ cells/mL were used as initial cell numbers. Cell cultures were diluted with a fresh medium every 48 h, and DMSO concentrations were adjusted accordingly. Differentiation into neutrophil-like cells was assessed via confocal imaging subsequent to 4',6-diamidino-2-phenylindole (DAPI) staining as well as by flow cytometry assay using PE-CD44 antibody (Moll *et al.*, 1998; Spring *et al.*, 1988). Granularity was also assessed via side-scattered light (SSC).

#### b) LPS-activated cells

HL-60 cells (8 x 10^6^) were transferred into 6-well plates and incubated overnight for LPS activation. The media was switched to 0.5% serum containing media and incubated for an additional 24 h. HL-60 cells were stimulated by 100 μg/mL LPS. Additionally 10 ng/μL phorbol-12-myristate-13-acetate (PMA) was added 5 h before LPS induction to enhance inflammation (Galarce *et al.*, 2008).

#### c) Globally methylated and demethylated cells

HL-60 (1 x 10^6^) cells were incubated with 5% (v/v) methanol for 2 days to increase methylation *in vitro* (Huang *et al.*, 2001). Also, 20 μM 5-aza-2'deoxycytidine (deazacytidine) was added into cell culture media for 72 h to reduce global methylation.

### Quantitative real-time PCR for exon 2 transcription analysis

Total RNA isolation from the established cell cultures (5 x 10^6^ cells approximately) was done by High Pure RNA Isolation Kit (Roche Diagnostics, Mannheim, Germany). High Capacity Reverse Transcription Kit (Applied Biosystems, Inc. Foster City, California) was used for cDNA synthesis.

Expression analyses were done using Power SYBR® Green Master Mix (Thermo Fisher Scientific Inc., Waltham, MA USA) and performed at StepOnePlus Real-Time PCR System (Applied Biosystems®). Primers listed in supplementary Table S4 were used for detecting different levels of *MEFV* transcripts of the cell culture models: The 1–3 primer, encompassing the junction of exons 1 and 3, amplifies *MEFV* transcripts without exon 2. The 2–3 primer, binding to the junction of exons 2 and 3, amplifies all other forms containing exon 2. Through these amplifications, we could acquire all possible *MEFV* transcripts with these two primers. GAPDH was used as a house-keeping gene.

The relative expression level was calculated using the ΔCT method. All reactions were done in duplicates (technical replicates) and were repeated three times (biological replicates). To compare the *MEFV*-d2 transcript ratio in cell culture models, and considering that the relative expression of both transcripts varies greatly in these cells, we normalized our data as follows:

$$\text{Ratio of d2 transcript}=\frac{\Delta CTd2}{\Delta CTd2+\Delta CTfl}$$

$$\text{Ratio of f1 transcript}=\frac{\Delta CTfl}{\Delta CTd2+\Delta CTfl}$$

### Region-specific methylation analysis of the cell culture models

DNA was isolated from the established cell cultures (5 x 10^6^ cells approximately) using DNA Isolation Kit for Cells and Tissues (Roche Diagnostics, Mannheim, Germany). Methylation analyses were performed with One-Step qMethyl Kit (Zymo Research, Irvine, CA, USA) using primers amplifying the *MEFV* CpG island, analyzed via qRT-PCR. Two reactions were setup as Test and Reference reactions: The Test reaction includes Methylation Sensitive Restriction Enzymes (MSREs) to cut at the methylated nucleotides, whereas the Reference reaction does not contain these enzymes. Therefore, the Test reaction samples are cut if methylated, creating smaller fragments, which result in lower Ct values.

The data was analyzed with qMethyl Calculator, which calculates the methylation ratio as follows: Percent methylation = 100 x 2^-ΔCt^.

where ΔCt is the average Ct value from the Test reaction minus the average Ct value from the Reference reaction.

### Pyrin localization analysis

Plasmid constructs containing CMV promoter and GFP tagged full-length cDNA sequence (pCMV6-AC-GFP-*MEFV*-fl) (Figure S1) and the cDNA sequence without the second exon of *MEFV* (pCMV6-AC-GFP-*MEFV*-d2) (Figure S2) were manufactured by Origene Technologies, Inc. (Rockville, Maryland, ABD). These constructs (2 μg) were transfected to 2 x 10^6^ HL-60 cells, neutrophil-like cells and LPS+PMA induced cells using Lonza Nucleofection Kit V (Amaxa, Cologne, Germany) with appropriate Nucleofector Program X-001 (X-01) for Nucleofector® I Device. For fluorescent staining and imaging, 24 hours after transfection, cells were fixed with methanol, and DAPI was used for cell nucleus imaging. Cells were visualized using laser confocal microscopy (Leica TCS SP2 SE, Wetzlar, Germany). Localization studies were performed with at least three transfections and the images were obtained with a 63 oil objective.

### Statistical analysis

Expression variations of *MEFV* transcripts, as well as methylation ratios were analyzed by using two-tailed unpaired t-test in Graphpad Prism (v. 6.0) software (GraphPad Software Inc, La Jolla, CA USA) and were reported as means and two-sided 95% confidence intervals.

## Results

### Analysis of splicing reporter assay of methylated *MEFV*-exon2

**Figure 1:**
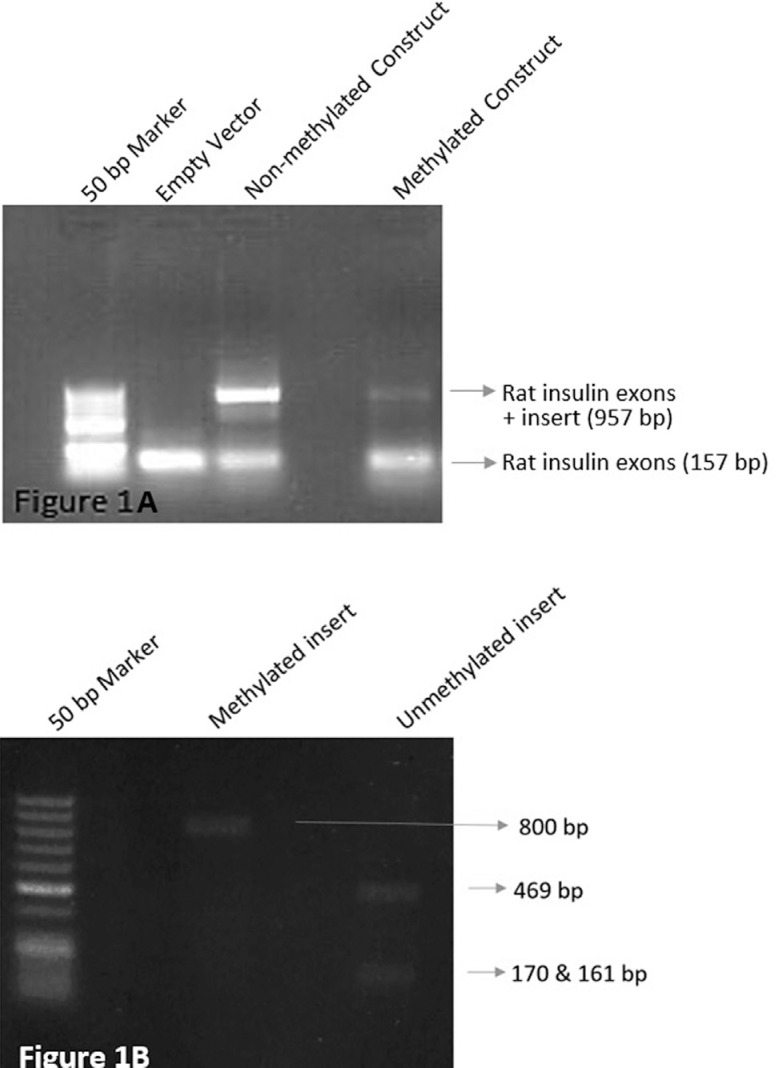
Agarose gel results of the splicing reporter experiments A) Agarose gel of PCR performed with primers specific to rat insulin exons. Methylation of the *MEFV* second exon leads to its splicing, which can be observed as a broader band at 157 bp, similar to the empty vector amplified with rat insulin exon primers. B) Agarose gel of the restriction enzyme analysis for confirmation of the methylation. The methylated construct is not cut, whereas non-methylated construct is cut by *Sma*I enzyme, which is sensitive to methylation.

We first tested whether methylation affects splicing by transfecting HL-60 cells with methylated and unmethylated constructs containing the 633 bp *MEFV* exon 2 plus the intron sequences, 800 bp in total, cloned in pSpliceExpress splicing vector. This vector allows tracing splicing events by means of its rat insulin exons splicing concurrently. We have shown that *MEFV* exon 2 is spliced when methylated (Figure 1), resulting in a smaller amplicon that lacks the second exon of *MEFV*.

### Splicing analysis of the cells' *MEFV* transcripts

Different cell culture inflammation models were established to analyze the effect of methylation on the exon 2 splicing of the cells' endogenous *MEFV* transcripts. To this end, HL-60 promyelotic cells were used as control groups and were induced with different agents: DMSO for neutrophil-like transformation, PMA and LPS for activation, methanol for methylation and deazacytidine for demethylation.

Differentiation of HL-60 cells into neutrophil-like cells was achieved as shown in DAPI staining (Figure S3), flow cytometry analysis using PE-CD44 antibody (Figure S4) as well as SSC results (Figure S5), given in supplementary data. CD44 is a cell surface glycoprotein involved in cell–cell interactions, cell adhesion and migration, and is expressed in different cell types including hematopoietic cells. DMSO-induced differentiation towards neutrophils causes downregulation of CD44 from the surface of cells compatible with a similar reduction in CD44 expression during normal granulopoiesis process (Moll *et al.*, 1998; Spring *et al.*, 1988).

Methanol is known as a toxic and mutagenic substance, generally used as a fixing agent in cell imaging. It has also been shown to increase genomic methylation by incorporating methyl residues into DNA (Huang *et al.*, 2001). In our methylation cell model, we also used 5% (vol/vol) methanol to increase global methylation levels in HL-60 cells.

**Figure 2:**
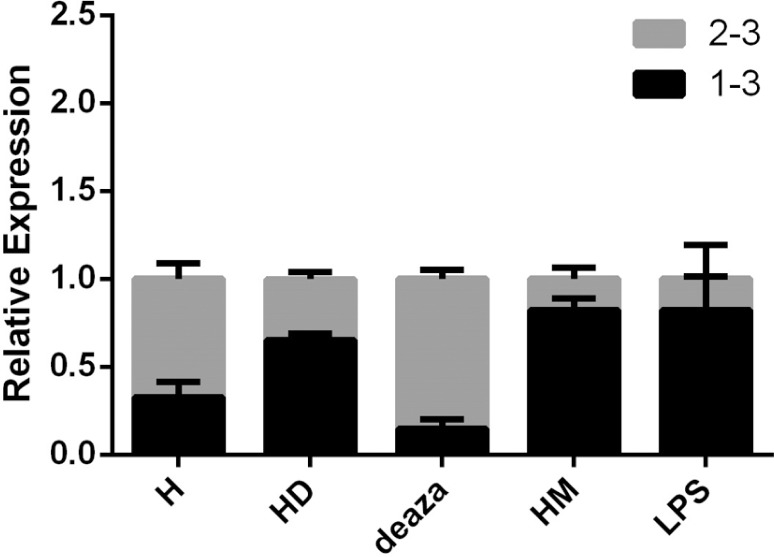
Relative expression results of different cell culture models. The 1-3 are exon 2 spliced transcripts' expressions and 2-3 exon 2 containing transcripts' expressions. H: untreated HL-60 cells; HD: HL-60 cells differentiated to neutrophil-like cells via DMSO; deaza: demethylated HL-60 cells using deazacytidine; HM: methylated HL-60 cells with methanol; LPS: HL-60 cells activated through PMA & LPS. The expression comparisons were significant with p < 0.0001 for H *vs.* HM, p = 0.0005 for H *vs.* HD, p = 0.0034 for H *vs.* LPS and p = 0.0126 for H *vs.* deaza (p < 0.05 was considered statistically significant, N = 4 for each cell model).

Expression studies were performed via two sets of primers amplifying cells endogenous *MEFV* exon 2-lacking and exon 2-containing transcripts and the data was normalized by dividing individual CTs to the total transcripts'. We observed that neutrophil-like cells (p = 0.0005, 2-fold increase), activated cells (p = 0.0034; 2.5-fold increase) and methylated cells (p < 0.0001; 2.5-fold increase) exhibit an increased *MEFV*-d2 transcript expression whereas demethylated cells (p = 0.0126; 1.7-fold decrease) exhibit a decreased level, compared to untreated HL-60 cells (Figure 2).

**Figure 3:**
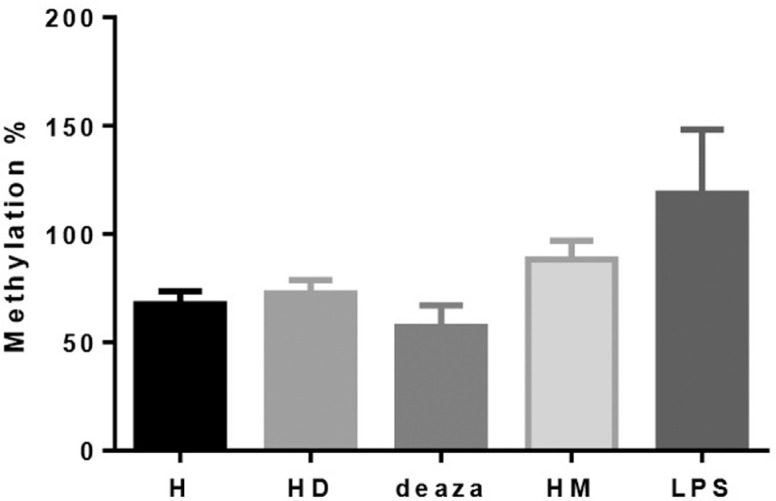
Average methylation levels of *MEFV* second exon in different cell culture models. H: untreated HL-60 cells; HD: HL-60 cells transformed to neutrophil-like cells via DMSO; deaza: demethylated HL-60 cells using deazacytidine; HM: methylated HL-60 cells with methanol; LPS: HL-60 cells activated through PMA & LPS. These methylation comparisons were non-significant, with a trend in H *vs.* HD (p = 0.4) and H *vs.* LPS (p = 0.053) (p < 0.05 was considered statistically significant, N = 3 for each cell model).

Analysis of induced and repressed global methylation studies in cell culture models confirmed that DMSO, PMA & LPS and methanol treatments increase, while deazacytidine decreases *MEFV* second exon methylation (Figure 3). The differences in methylation level were not statistically significant, with a positive trend in HL-60 cells treated with DMSO (p = 0.4) and HL-60 cells treated with PMA & LPS (p = 0.053).

### Cellular localization of pyrin and its isoform pyrin-d2

The localization of full-length pyrin and exon 2-lacking pyrin isoform (pyrin-d2) were investigated in HL-60 cells along with neutrophil-like cells via confocal microscopy, since our findings imply that *MEFV*-d2 transcript is increased in these cells.

**Figure 4:**
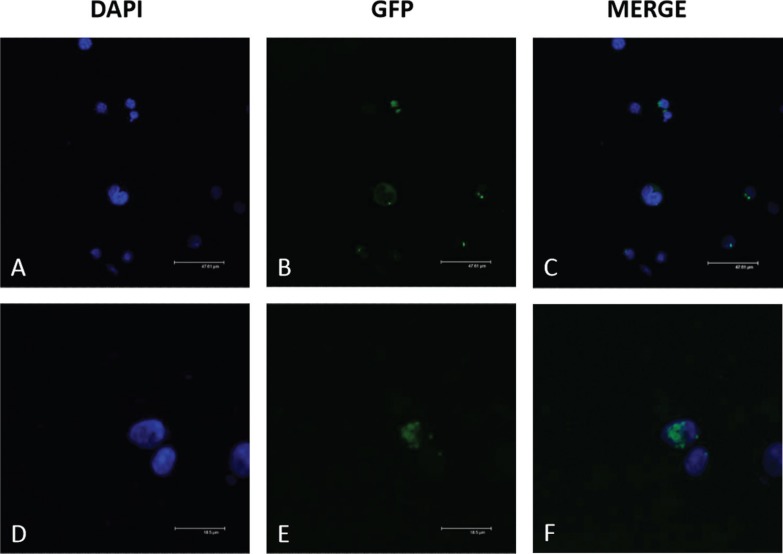
Localization of recombinant *MEFV* proteins in untreated HL-60 cells. *MEFV*-fl-GFP and *MEFV*-d2-GFP constructs were transfected via Nucleofection and the localization of their products was analyzed using confocal microscopy. The transfection efficiency was 67% for *MEFV*-fl and 58% for *MEFV*-2d. A) DAPI staining of HL-60 cells transfected with *MEFV*-fl-GFP; B) GFP visualization of *MEFV*-fl-GFP; C) Merged image of A and B; D) DAPI staining of HL-60 cell transfected with *MEFV*-d2-GFP; E) GFP visualization of *MEFV*-d2-GFP; F) Merged image of D and E.

**Figure 5:**
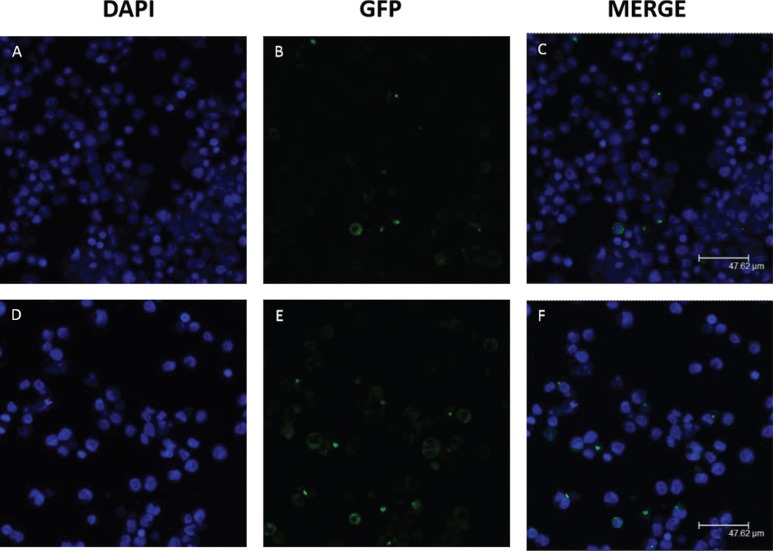
Localization of recombinant *MEFV* proteins into neutrophil-like cells. 5 x 10^4^ cells/mL were incubated with DMSO for 6 days. *MEFV*-fl-GFP and *MEFV*-d2-GFP constructs were transfected via Nucleofection and the localization of their products was analyzed using confocal microscopy. The transfection efficiency was 50% for *MEFV*-fl and 72% for *MEFV*-2d. A) DAPI staining of DMSO induced HL-60 cell transfected with *MEFV-*fl-GFP; B) GFP visualization of *MEFV*-fl-GFP; C) Merged image of A and B; D) DAPI staining of DMSO induced HL-60 cell transfected with *MEFV*-d2-GFP; E) GFP visualization of *MEFV*-d2-GFP; F) Merged image of D and E.

In untreated HL-60 controls, the *MEFV*-fl-GFP protein was localized in the cytoplasm and *MEFV*-d2-GFP was localized in the nucleus (Figure 4). In contrast, in neutrophil-like cells, both *MEFV*-fl-GFP and *MEFV*-d2-GFP products were localized in the cytoplasm (Figure 5).

## Discussion

Alternative splicing causes the production of protein isoforms with differential subcellular localization, which may lead to altered functions (Stamm *et al.*, 2005; Hughes, 2006). More than 90% of human genes are subjected to alternative splicing, which is an evolutionarily conserved mechanism, ensuring proteomic diversity (Ast, 2004; Pan *et al.*, 2008). Co-transcriptional splicing enables the formation of alternative transcripts through epigenetic regulation of the chromatin (Iannone and Valcárcel, 2013). Epigenomic studies have illustrated that CG dinucleotides were more abundant on exonic sites than intronic sites, and 3' and 5' splice sites were mostly methylated (nearly 100%) compared to surrounding CG rich regions (Gelfman *et al.*, 2013).

Previously, we showed that *MEFV* exon 2 methylation levels of patients were slightly but significantly higher than the levels of controls (p = 0.049) (Kirectepe *et al.*, 2011b). Furthermore, we observed a negative correlation between methylation and expression in all groups (r=–0.29, p = 0.041), which was more accentuated in the patient group (r=–0.36, p = 0.035). Here, we proposed a model where the methylation of *MEFV* exon 2 is related to its alternative splicing and expression. To test that hypothesis, *MEFV* exon 2 including approximately 80 nucleotides of its flanking introns was cloned into a splicing reporter vector, which proved that transfected *MEFV* exon 2 is spliced when methylated. Then, different cell culture models were constructed to confirm this finding with endogenous *MEFV* expression: HL-60 cells were used as controls and DMSO induced cells, activated cells trough PMA and LPS, methylated cells with methanol, demethylated cells via deazacytidine constituted the induced group where the expression level of *MEFV* exon 2-containing and lacking-transcripts were analyzed. The results indicated that methylation increased the expression of *MEFV*-d2 transcript, along with neutrophil transformation and activation with PMA & LPS, contrary to demethylated cells in which it was decreased. These findings suggest that methylation might play a role in the splicing of the second exon. The methylation levels of the CpG island of these cell culture models were further analyzed (Figure 3). Although the differences were not statistically significant, they exhibited similar increased methylation patterns, implying that methylation increases in inflammatory-like conditions.

An increasing number of studies are pointing to the involvement of DNA methylation in the regulation of alternative splicing. A genome wide study performed in mouse retina and brain showed that differently methylated regions regulate alternative splicing in a tissue-specific manner (Wan *et al.*, 2013). Another genome-wide study on honey bees, in which the authors inhibited the expression of Dnmt3 (DNA (cytosine-5)-methyltransferase 3), changed the alternative splicing pattern due to the decrease in methylation levels (Li-Byarlay *et al.*, 2013). A recent study proposed that alternative splicing of sarcomeric gene *Myh7* may be linked to the cardiac epigenome, which may lead to disease formation (Vujic *et al.*, 2015). Absence of the *MEFV* exon 2-deleted form in mice and rats, which do not contain a CpG island on the *MEFV* gene (Papin *et al.*, 2000), also points towards the possible role of DNA methylation in alternative splicing, and also strengthens our hypothesis.

Several mechanisms have been proposed in attempting to explain the role of epigenetic modifications in alternative splicing. Some studies have shown that methylation leads to the inclusion of alternative exons. For example, MeCP2 (methyl-CpG binding protein 2) and HP1 (heterochromatin protein 1) are proteins found to participate in exon retention in the presence of DNA methylation (Yearim *et al.*, 2015). Adversely, another protein, CTCF, was found to play a role in the recognition of a weak exon signal in the *CD45* gene in the absence of DNA methylation (Shukla *et al.*, 2011). Although our research on the CTCFBSDB database, a database for CTCF binding sites, has shown the absence of CTCF in the *MEFV* gene in HL-60 cells, the CTCF prediction tool on the same site predicts a binding site 35 bp from the exon recognition consensus sequence. As alternative splicing is regulated via splice site strength, and, thus, stronger sites increase the inclusion of alternative exons (Schwartz *et al.*, 2009), recognition of weak splice sites via reducing RNA PolII elongation rate is crucial for the retention of subjected exons. Methylation may also simply block *cis*-acting sequences where RNA-binding proteins bind to enhance the inclusion level. We found many putative exonic splicing enhancer sites containing a CG dinucleotide, which could be blocked by methylation (Human Splicing Finder). Therefore, differential methylation of specific sites within a CpG island may be responsible for exon inclusion levels, rather than methylation of a specific region. It would be very informative to perform an analysis of a potential protein, like CTCF, slowing down the elongation rate of the RNA PolII and thus enabling the recognition of the *MEFV* exon 2 signal, leading to its inclusion. This would also suggest methylation of its specific binding site, which will be further analyzed.

Point mutations/variations are also known to cause alternative splicing. Rittore *et al.* (2014) showed three variations [rs4149570(c.-610G > T), rs767455(c.36A > G,pPro12Pro), rs1800692(c.473-33C > T)] in the promoter, exon 1 and intron 2, respectively, of TNFRSF1A gene enhance the splicing of exon 2 *in vitro*. These variations appeared to have a role in the pathogenesis of TRAPS disease (Rittore *et al.*, 2014). Furthermore, in another study, Tone *et al.* (2011) suggested that c.910G > A variant (rs75977701) leads to the skipping of MEFV exon 2. This rare variant, which has a Minor Allele Frequency (MAF) of T = 0.0074/37 in the Thousands Genomes Project, was absent in our FMF cohort. In Japan, where this variant is more frequently encountered, its frequency in FMF patients is 0.9% (Kishida *et al.*, 2014). This is in contradiction with our finding of higher MEFV-d2 transcript levels in FMF patients compared to healthy controls (Kirectepe *et al.*, 2011a), suggesting a different mechanism for the splicing of exon 2, at least for the Turkish population.

Since many diseases are shown to be caused by abnormal localization of proteins, the localization differences of GFP tagged full-length pyrin (pyrin-fl) and exon 2 spliced forms (pyrin-d2) were also analyzed in inflammation related cell culture models. Both *MEFV*-fl-GFP and *MEFV*-d2-GFP proteins were found to be localized in the cytoplasm of DMSO-treated neutrophil-like cells (Figure 4). On the other hand, in non-treated HL-60 cells, the product of *MEFV*-fl-GFP construct was cytoplasmic, in contrast with the *MEFV*-d2-GFP product present in the nucleus (Figure 5). This type of experiment requires a western blot confirmation in cytoplasmic *vs.* nuclear fractions. However, only a maximum of 40% of HL-60 cells differentiated into neutrophil-like cells, and the transfection efficiency was not constant among the experiments. Thus western blot studies had inconsistencies in these *in vitro* models. Therefore, we increased the repeats of our confocal experiments to obtain reliable results. The 14.3.3 proteins, potent anti-apoptotic factors that control intracellular signaling, cell cycle and apoptosis, interact with pyrin-fl but not pyrin-d2 through three serine residues located in exon 2. Jéru *et al.* (2005) showed that this interaction caused full-length pyrin to be retained in the cytoplasm. Furthermore, the lack of interaction with pyrin-d2 isoform resulted in protein translocation to the nucleus. Thus, cell-specific post-translational processing and/or protein–protein interactions, may ultimately determine subcellular localization relating to pathological functions in different cell types. Although alternative splicing of exon 2 creates a nuclear localization signal, it does not correspond to known NLS motifs. Exon 1-3 junction encodes a domain, which is necessary but not sufficient to target the spliced form to nucleus. Thus, this domain may be required for the activity of another NLS-like motif located elsewhere in the spliced form. We and other authors previously suggested that pyrin-d2 may have a role as a transcription factor, and its function may be impaired during inflammation (Kirectepe *et al.*, 2011a). Thus, understanding the proteins that interact with the exon 2-deleted but not the full-length pyrin protein and their functions may lead to identification of a mechanism involved in nuclear import and inflammatory diseases.

Several other studies analyzed similar features: Tidow *et al.* (2000) studied the localization of full-length pyrin in COS-1 and found it to be cytoplasmic. They also found that DMSO induction increases the full-length expression in HL-60 cells but they did not investigate the expression of exon 2 spliced transcript. Diaz *et al.* (2004) induced the expression of exon 2 spliced form by LPS in synovial fibroblasts and monocyte-enriched PBLs from patients; however, they found it to be still two times less abundant than the full-length form. We had opposite results with HL-60 promyelotic cells, which are known to be neutrophilic precursors, suggesting that the expression of spliced transcript may change with harmful stimuli, since neutrophils are the first responders in cases of inflammation. Cazeneuve *et al.* (2003) reported pyrin-d2 to be in the nucleus of HeLa cells; nevertheless, they also found that it interacts with ASC in the cytoplasm. In a recent study, pyrin was shown to co-localize with actin in HL-60 cells (Akkaya-Ulum *et al.*, 2015). Knowing that the overexpression of recombinant products transfected to cells in a transient manner could disrupt physiological pathways for protein transport (Kremmidiotis *et al.*, 1999), it could be of interest to generate an antibody specific to exon 2 spliced form to detect the localization of native protein in different cells via confocal microscopy and western blot analyses.

Despite the fact that FMF is reported to be a recessively inherited disease, 15 to 25% of patients from locations where FMF is less prevalent present no pathogenic variations in the *MEFV* gene. A modifier gene has been suggested as a primary alternative, and studies were conducted to identify another gene or locus having an epistatic interaction with *MEFV*. Touitou *et al.* (2001) proposed *MICA* as a modifier gene in FMF; however, they could not find a significant result for affected patients from different ethnic origins. Hence, any disruption leading to truncated pyrin formation may have a role in the pathology of FMF. Our findings clearly show the differences between undifferentiated and differentiated cells. Research should be conducted with patients in active inflammation and relapse periods and healthy controls to further understand the exact mechanism. However, methylation leading to the splicing of *MEFV* second exon, and consequently to a protein that has an aberrant localization, may explain the pathogenesis of FMF without the *MEFV* pathogenic variants.

## Supplementary material

The following online material is available for this article:

Table S1 - Primers used for the amplification of pSpliceExpress insert sequence.

Table S2 - Primers used for adding attb 1&2 recombination sites.

Table S3 - Rat insulin primers.

Table S4 - Primers for quantitative real-time PCR.

Figure S1 - DMSO induction of HL-60 cells.

Figure S2 - CD44 expression analysis of undifferentiated and differentiated HL-60 cells.

Figure S3 - Granularity analysis of undifferentiated and differentiated HL-60 cells via side-scattered light (SSC).

Figure S4 - pCMV6-AC-GFP-MEFV-FL (OriGene) construct.

Figure S5 - Granularity analysis of undifferentiated and differentiated HL-60 cells via side-scattered light (SSC).
