# Supplementary material for: Alternatively spliced MEFV transcript lacking exon 2 and its protein isoform pyrin-2d implies an epigenetic regulation of the gene in inflammatory cell culture models
Source: Genet Mol Biol. 2017 Aug 31;40(3):688–97. doi: 10.1590/1678-4685-GMB-2016-0234 (PMC5596369; doi:10.1590/1678-4685-GMB-2016-0234)
Supplement: Supplementary file 2 [file 1415-4757-gmb-1678-4685-GMB-2016-0234-Suppl02.pdf]

Genetics and Molecular Biology, 40, 3 (2017)

Supplementary material to "Alternatively spliced MEFV transcript lacking exon 2 and its protein isoform pyrin-2d implies an epigenetic regulation of the gene in inflammatory cell culture models"

Table S2. Primers used for adding attb 1&2 recombination sites

Oligonucleotide primer name	Sequence
attB1 Forward	5'-GGGGACAAGTTTCTACAAAAAAGCAGGCTCAAGGGGATTCTCTCTCCTCT-3'
attB2 Reverse	5'- GGGGACCACTTTGTACAAGAAAGCTGGGTTGGGATTACAGGCATGAGCT-3'
