# Supplementary material for: Alternatively spliced MEFV transcript lacking exon 2 and its protein isoform pyrin-2d implies an epigenetic regulation of the gene in inflammatory cell culture models
Source: Genet Mol Biol. 2017 Aug 31;40(3):688–97. doi: 10.1590/1678-4685-GMB-2016-0234 (PMC5596369; doi:10.1590/1678-4685-GMB-2016-0234)
Supplement: Supplementary file 4 [file 1415-4757-gmb-1678-4685-GMB-2016-0234-Suppl04.pdf]

Genetics and Molecular Biology, 40, 3 (2017)

Supplementary material to "Alternatively spliced MEFV transcript lacking exon 2 and its protein isoform pyrin-2d implies an epigenetic regulation of the gene in inflammatory cell culture models"

Table S4. Primers for quantitative real-time PCR. Primers were designed at Universal Probe Library website of Roche (www.roche-applied- science.com/sis/rtpcr/upl/index.jsp?id=UP030000).

**Oligonucleotide primer name**	**Oligonucleotide primer sequence**
MEFV 1-3 Forward	5'-CAT TCA GGG AAG GCC ACC AG-3'
MEFV 1-3 Reverse	5'-TTC CTT TCA TGG GAG TCC TG-3'
MEFV 2-3 Forward	5'-GAA ATC CAG AAC ATT CGG TCA-3'
MEFV 2-3 Reverse	5'-ACC GTC AAC TGG GTC TCC TT-3'
GAPDH	TaqMan® GAPDH Control Reagents (human) (Life Technologies) Primers
