# Supplementary material for: Alternatively spliced MEFV transcript lacking exon 2 and its protein isoform pyrin-2d implies an epigenetic regulation of the gene in inflammatory cell culture models
Source: Genet Mol Biol. 2017 Aug 31;40(3):688–97. doi: 10.1590/1678-4685-GMB-2016-0234 (PMC5596369; doi:10.1590/1678-4685-GMB-2016-0234)
Supplement: Supplementary file 5 [file 1415-4757-gmb-1678-4685-GMB-2016-0234-Suppl05.pdf]

Genetics and Molecular Biology, 40, 3 (2017)

Supplementary material to "Alternatively spliced MEFV transcript lacking exon 2 and its protein isoform pyrin-2d implies an epigenetic regulation of the gene in inflammatory cell culture models"

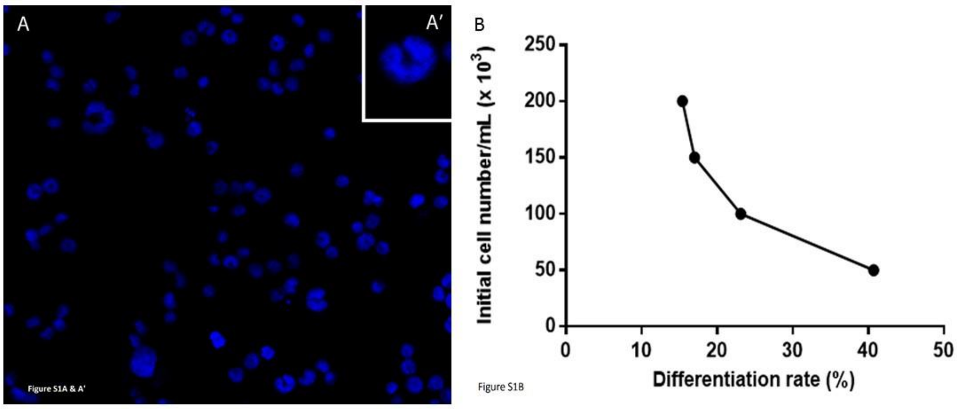

Figure S1. DMSO induction of HL-60 cells. A) DAPI staining of differentiated HL-60 cells. Over 40% of HL-60 cells were shown to be differentiated to neutrophil-like cells after 6-day incubation with 1.75% DMSO. Insert A', segmented nucleus, typical feature of neutrophils; B) differentiation rate with regard to incubation length and initial cell number. Each dot represents the mean of different experiments. Differentiation increases with decreasing initial cell amount and the optimal cell number is determined as 5×104 cells/mL.
