# Supplementary material for: Alternatively spliced MEFV transcript lacking exon 2 and its protein isoform pyrin-2d implies an epigenetic regulation of the gene in inflammatory cell culture models
Source: Genet Mol Biol. 2017 Aug 31;40(3):688–97. doi: 10.1590/1678-4685-GMB-2016-0234 (PMC5596369; doi:10.1590/1678-4685-GMB-2016-0234)
Supplement: Supplementary file 6 [file 1415-4757-gmb-1678-4685-GMB-2016-0234-Suppl06.pdf]

Genetics and Molecular Biology, 40, 3 (2017)

# Supplementary material to "Alternatively spliced MEFV transcript lacking exon 2 and its protein isoform pyrin-2d implies an epigenetic regulation of the gene in inflammatory cell culture models"

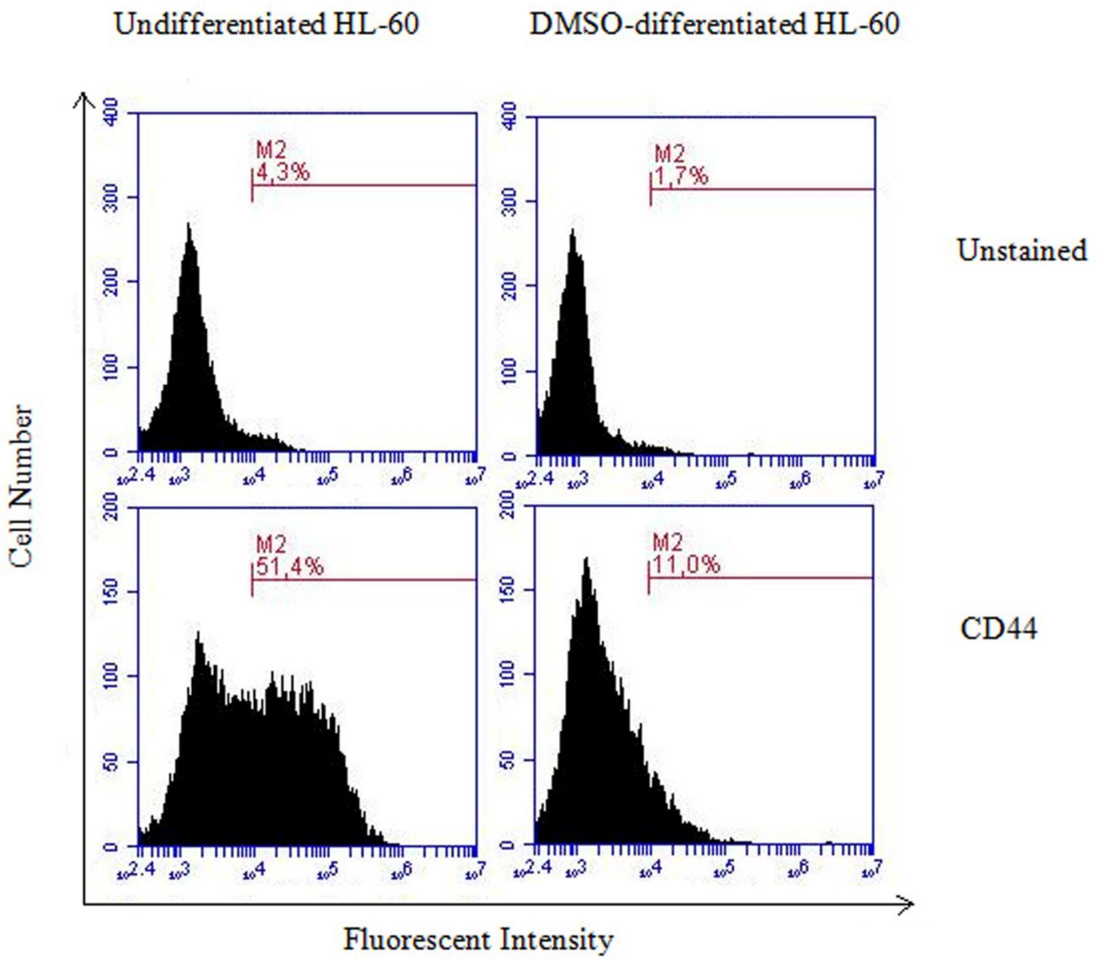


**Figure S2 -** CD44 expression analysis of undifferentiated and differentiated HL-60 cells. Comparison of CD44 levels of untreated and DMSO-treated cells via flow cytometry analysis showed that DMSO treated cells have lower CD44 expression compared to untreated cells, consistent with normal granulopoiesis process.
