# Supplementary material for: Alternatively spliced MEFV transcript lacking exon 2 and its protein isoform pyrin-2d implies an epigenetic regulation of the gene in inflammatory cell culture models
Source: Genet Mol Biol. 2017 Aug 31;40(3):688–97. doi: 10.1590/1678-4685-GMB-2016-0234 (PMC5596369; doi:10.1590/1678-4685-GMB-2016-0234)
Supplement: Supplementary file 7 [file 1415-4757-gmb-1678-4685-GMB-2016-0234-Suppl07.pdf]

Genetics and Molecular Biology, 40, 3 (2017)

Supplementary material to "Alternatively spliced MEFV transcript lacking exon 2 and its protein isoform pyrin-2d implies an epigenetic regulation of the gene in inflammatory cell culture models"

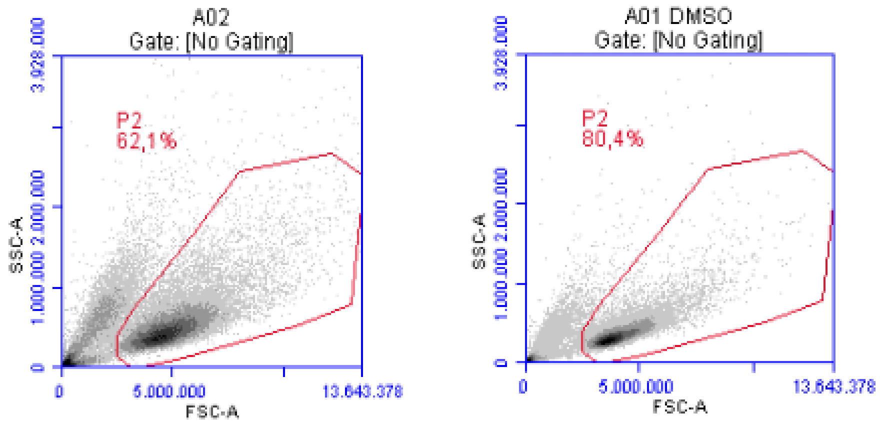

Figure S3. Granularity analysis of undifferentiated and differentiated HL-60 cells via side-scattered light (SSC). DMSO-treated cells show higher granularity than untreated cells, confirming our previous analysis.
